# New multiple sclerosis lesion segmentation and detection using pre-activation U-Net

**DOI:** 10.3389/fnins.2022.975862

**Published:** 2022-10-26

**Authors:** Pooya Ashtari, Berardino Barile, Sabine Van Huffel, Dominique Sappey-Marinier

**Affiliations:** ^1^Department of Electrical Engineering (ESAT), STADIUS Centre for Dynamical Systems, Signal Processing and Data Analytics, KU Leuven, Leuven, Belgium; ^2^CREATIS (UMR 5220 CNRS – U1294 INSERM), Université Claude Bernard Lyon 1, Université de Lyon, Villeurbanne, France

**Keywords:** multiple sclerosis, new lesions, segmentation, U-Net, pre-activation

## Abstract

Automated segmentation of new multiple sclerosis (MS) lesions in 3D MRI data is an essential prerequisite for monitoring and quantifying MS progression. Manual delineation of such lesions is time-consuming and expensive, especially because raters need to deal with 3D images and several modalities. In this paper, we propose Pre-U-Net, a 3D encoder-decoder architecture with pre-activation residual blocks, for the segmentation and detection of new MS lesions. Due to the limited training set and the class imbalance problem, we apply intensive data augmentation and use deep supervision to train our models effectively. Following the same U-shaped architecture but different blocks, Pre-U-Net outperforms U-Net and Res-U-Net on the MSSEG-2 dataset, achieving a Dice score of 40.3% on new lesion segmentation and an F_1_ score of 48.1% on new lesion detection. The codes and trained models are publicly available at https://github.com/pashtari/xunet.

## 1. Introduction

Multiple sclerosis (MS) is a common chronic, autoimmune demyelinating disease of the central nervous system (CNS), which causes inflammatory lesions in the brain, particularly in white matter (WM). Multi-parametric MRI is widely used to diagnose and assess MS lesions in clinical practice. Particularly, FLuid Attenuated Inversion Recovery (FLAIR) images provide high contrast for white matter lesions appearing as high-intensity regions. It is highly relevant to monitor lesion activities, especially the appearance of new lesions and the enlargement of existing lesions, for several purposes, including prognosis and follow-up. More specifically, lesional changes between two longitudinal MRI scans from an MS patient are the most important markers for tracking disease progression and inflammatory changes. To this end, the accurate segmentation of new lesions is an essential prerequisite to quantifying lesional changes and measuring features, such as new lesion volumes and locations. However, manual delineation of such lesions is tedious, time-consuming, and expensive, especially because experts need to deal with 3D images and several modalities; therefore, accurate computer-assisted methods are needed to automatically perform this task.

**Figure 1:**
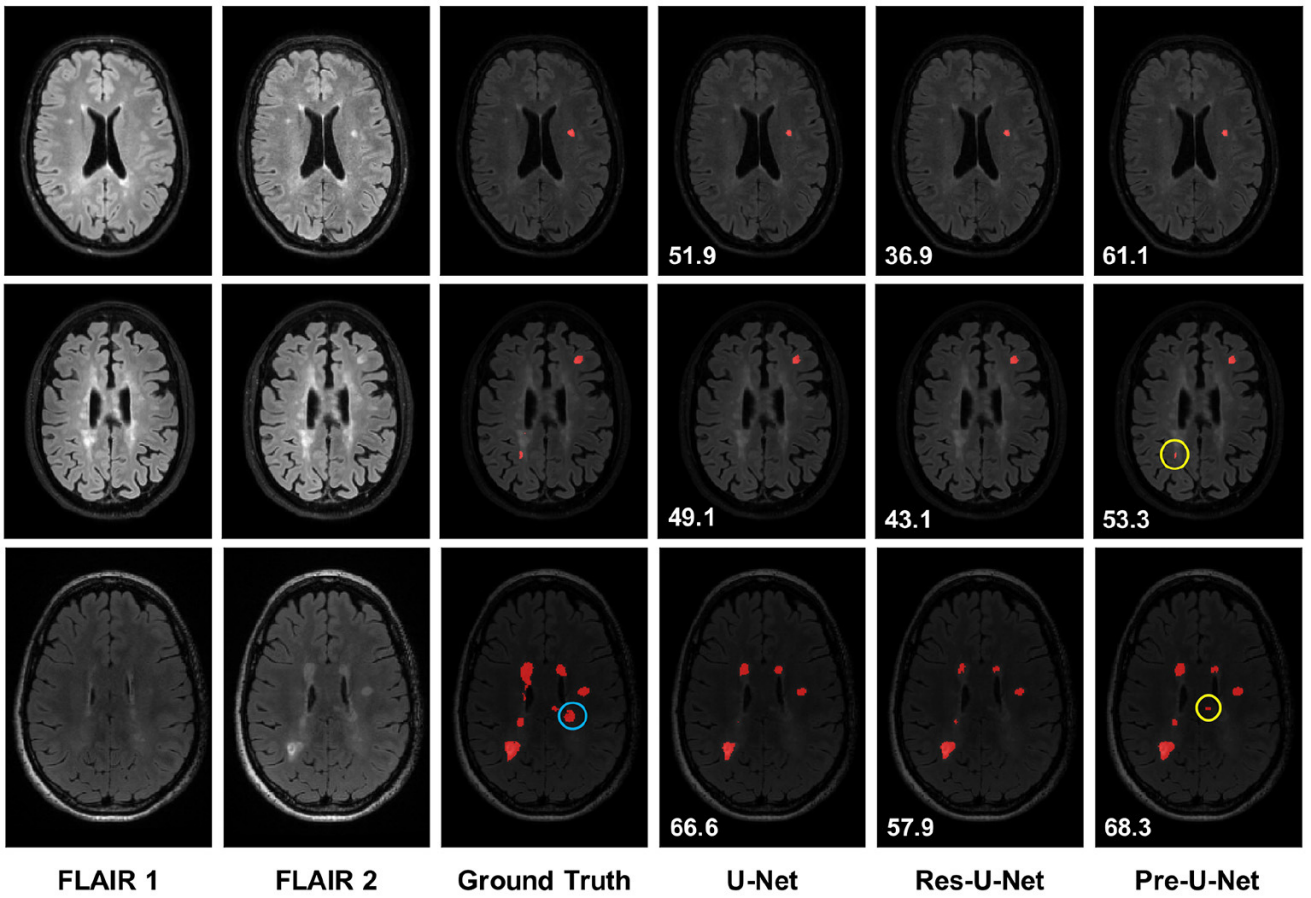
Qualitative results on new MS lesion segmentation. The three examples are from three different patients in the test set. The new lesions are shown in red in the segmentation maps. The new lesions circled in yellow (rows 2-3 and column 6) are successfully detected only by Pre-U-Net, while the new lesion circled in blue (row 3 and column 3) is not captured by any of the models, representing a very difficult case. The patient-wise Dice score for each example is displayed on the segmentation map.

Longitudinal MS lesion segmentation, however, remains very challenging since MS images often change subtly over time within a patient, and new lesions can be very small although they vary dramatically in shape, structure, and location across patients. The MSSEG-2 MICCAI 2021 challenge (Confavreux et al., 1992; Vukusic et al., 2020) aims to develop effective data-driven algorithms for the segmentation of new MS lesions by providing a dataset of 40 pairs of 3D FLAIR images acquired at two different time points (with varying intervals) and registered in the intermediate space between the two time points. For each pair, new lesions are manually annotated by multiple raters, and the consensus ground truths are obtained through a voxel-wise majority voting (see Figure 1).

Over the past decade, convolution neural networks (CNNs) with an encoder-decoder architecture, known as U-Net (Ronneberger et al., 2015), have dominated medical image segmentation. In contrast to a hand-crafted approach, U-Net can automatically learn high-level task-specific features for MS lesion segmentation. This work extends our previous effort (Ashtari et al., 2021a) in the MSSEG-2 and proposes Pre-U-Net, a 3D U-Net architecture with pre-activation residual blocks (He et al., 2016a,b), for segmenting new MS lesions. We use deep supervision (Lee et al., 2015) and perform intensive data augmentation to effectively train our models. In contrast to the existing methods, our models directly segment new MS lesions on longitudinal 3D FLAIR images in an end-to-end fashion in contrast to the common two-step approach, where cross-sectional segmentation is first performed individually for each time point, and new lesions are then extracted by comparing the longitudinal segmentation maps and applying further post-processing. Depending on the metric used, the MSSEG-2 challenge has four leaderboards. Our Pre-U-Net model achieved competitive scores, and our team, LYLE, was ranked first in two of the leaderboards among 30 participating teams in the challenge.

The rest of this paper is organized as follows: Section 2 briefly reviews relevant semantic segmentation techniques. Section 3 presents our approach to longitudinal MS lesion segmentation. Experiments are presented in Section 5. We conclude this paper in Section 6.

## 2. Related work

Over the past few years, considerable efforts have been made in the development of fully convolutional neural networks for semantic segmentation. Encoder-decoder architectures, in particular U-Net (Ronneberger et al., 2015) and its variants, are dominant in the segmentation of brain lesions. nnU-Net (Isensee et al., 2019) makes minor modifications to the standard 3D U-Net (Çiçek et al., 2016), automatically configuring the key design choices. It has been successfully applied to many medical image segmentation tasks, including longitudinal MS lesion segmentation (Isensee et al., 2020). McKinley et al. (2018) proposed an architecture, in which dense blocks (Huang et al., 2017) of dilated convolutions are embedded in a shallow encoder-decoder network. Myronenko (2019) proposed a U-Net-style architecture with a heavier encoder but a lighter decoder for brain tumor segmentation, taking a variational auto-encoder (VAE) approach by adding a branch to the encoder endpoint. Ashtari et al. (2021b) proposed a lightweight CNN for glioma segmentation, with low-rank constraints being imposed on the kernel weights of the convolutional layers in order to reduce overfitting. Aslani et al. (2019) proposed a deep architecture made up of multiple branches of convolutional encoder-decoder networks that perform slice-based MS lesion segmentation. La Rosa et al. (2020) proposed a U-Net-like model, to automatically segment cortical and white matter lesions based on 3D FLAIR and MP2RAGE images. These works and most of the MS research in medical imaging have focused on the cross-sectional segmentation of lesions, while only a few efforts have been made to detect and segment new lesions on longitudinal MRI scans. For example, Nills et al. (2020) proposed a two-path CNN jointly processing two FLAIR images from two time points to address longitudinal segmentation of new and enlarged lesions. In contrast, this paper proposes a single-path U-shaped architecture whose input is the 2-channel image constructed simply by concatenating two longitudinal FLAIR images which are co-registered.

## 3. Method

In this section, we describe the proposed encoder-decoder architecture, called Pre-U-Net, and its building blocks.

### 3.1. Overall architecture

**Figure 2:**
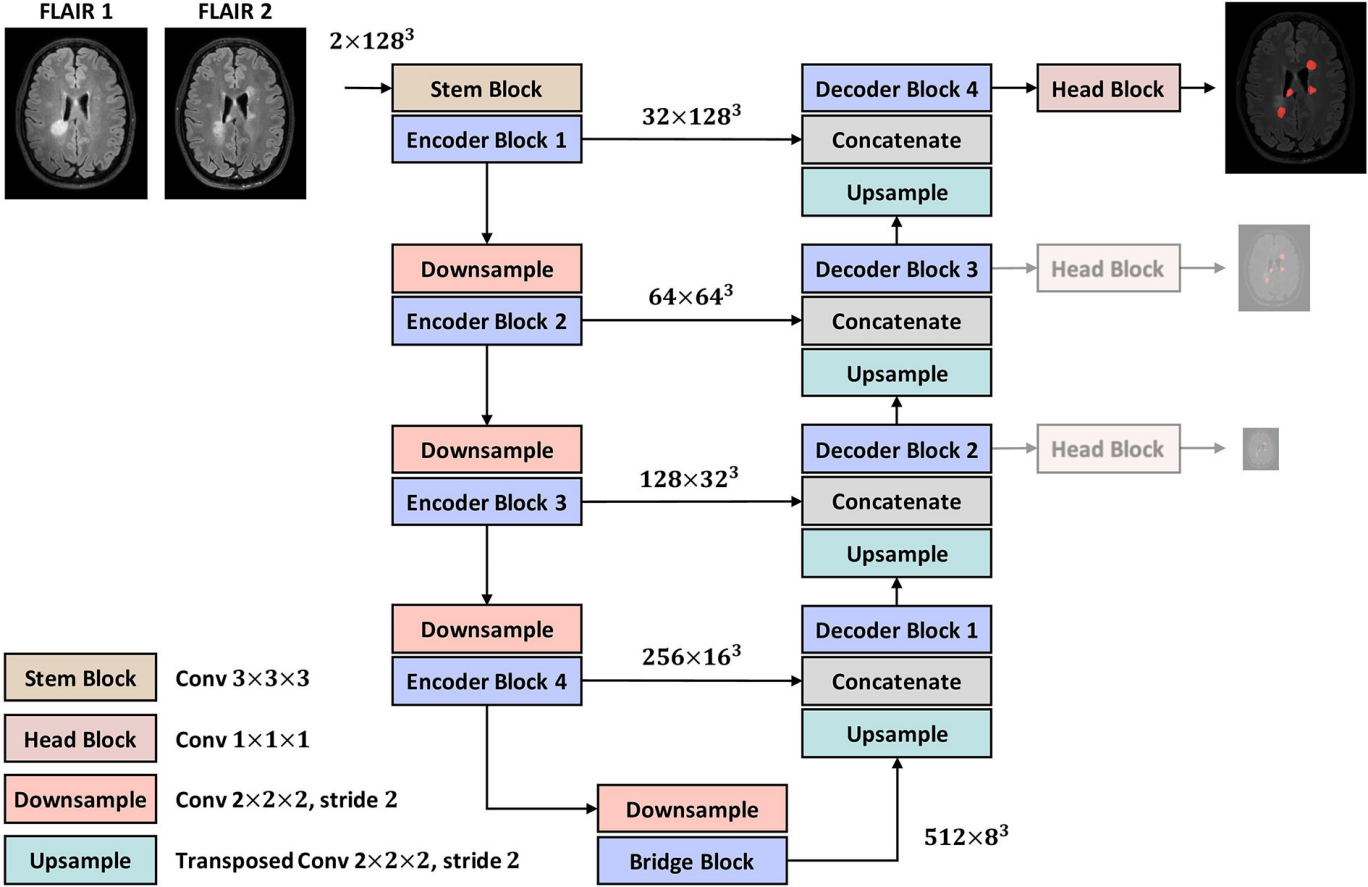
The proposed encoder-decoder architecture. The two lower-resolution auxiliary maps are only used in the training phase as deep supervisions.

The overall architecture, as shown in Figure 2, follows a U-Net-like style made up of encoder and decoder parts. A 3 × 3 × 3 convolution is used as the stem layer. The network takes a 2-channel image of size 128 × 128 × 128 and outputs a *probability map* with the same spatial size. The network has 4 levels, at each of which in the encoder (decoder), the input tensor is downsampled (upsampled) by a factor of two while the number of channels is doubled (halved). Downsampling and upsampling are performed *via* strided convolution and transposed convolution, respectively. The kernel size of all downsamplers and upsamplers is 2 × 2 × 2. We use deep supervision at the three highest resolutions in the decoder, applying pointwise convolutions (head blocks) to get three auxiliary logit tensors.

### 3.2. Baseline models

Depending on which block is used, we build and compare three baselines: (i) U-Net, (ii) Res-U-Net, and (iii) Pre-U-Net. All these variants follow the same overall architecture as explained in Section 3.1 but differ in their encoder/decoder blocks. The block for each model is detailed in the following.

#### 3.2.1. U-Net block

**Figure 3:**
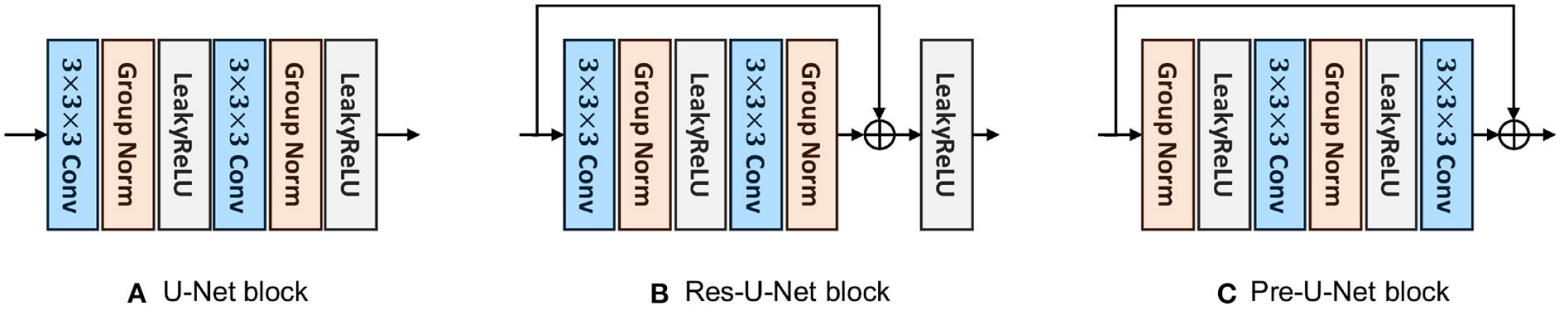
The proposed blocks. **(A)** U-Net block. **(B)** Res-U-Net block. **(C)** Pre-U-Net block.

The U-Net block used here is similar to that of nnU-Net (Isensee et al., 2019) except for some minor modifications. As shown in Figure 3A, this block is composed of two convolutional layers with kernel sizes of 3 × 3 × 3. A Group Normalization (Wu and He, 2018) layer (with a group size of 8) comes after each convolutional layer and before LeakyReLU activation.

#### 3.2.2. Res-U-Net block

Inspired by the basic ResNet block (He et al., 2016a), a Res-U-Net block is, as shown in Figure 3B, similar to U-Net block except that a shortcut connection is used between the last Group Normalization layer and the last LeakyReLU activation. A pointwise convolution (i.e., a kernel size of 1 × 1 × 1) may be used in the shortcut connection to match the input dimension with the output dimension of the residual mapping. As investigated by He et al. (2016a), residual connections have been proven effective to avoid vanishing/exploding gradients and speed up the convergence, especially in very deep networks.

#### 3.2.3. Pre-U-Net block

Similar to the pre-activation residual block (He et al., 2016b), a pre-U-Net block consists of two convolutional layers with kernel sizes of 3 × 3 × 3, with LeakyReLU activation coming before each convolutional layer and after Group Normalization (with a group size of 8). Note that the pre-U-Net block, in contrast to U-Net and Res-U-Net blocks, starts with normalization, applying convolution-activation-normalization in reverse order (see Figure 3C). He et al. (2016b) suggest that such a pre-activation design together with identity mappings as the shortcut connections makes information propagate more smoothly than the post-activation design (which is used in the basic ResNet block). Through ablation experiments, they show that the pre-activation design reduces overfitting more significantly, meaning that it leads to slightly higher training loss at convergence but lower test error compared to the post-activation design.

## 4. Experiments

All the models are implemented using PyTorch (Paszke et al., 2019) and PyTorch Lighting (Falcon, 2019) frameworks and trained on NVIDIA P100 GPUs. We evaluate the performance of Pre-U-Net for MS lesion segmentation on the MSSEG-2 dataset. We follow the same training workflow in all the experiments. In the following, we first provide the details of this workflow, then present the evaluation protocol and the results.

### 4.1. Setup

#### 4.1.1. Data

A total of 40 and 60 MS patients are represented in the MSSEG-2 training and test set, respectively. For each patient, two longitudinal 3D FLAIR images are acquired at different time intervals (e.g., 1 year, 3 years) and registered in the intermediate space between the two time points. New lesions that a patient developed between the two time points were manually delineated by multiple raters, and the consensus ground truths were obtained through a voxel-wise majority voting (see Figure 1). The training (test) set includes images that have no new lesions since, in real clinical practice, many patients under treatment do not develop any new lesions during the time interval. Further details on the MSSEG-2 dataset are reported in Table 1. Note that both the training and test sets were fixed across our experiments as well as for all the challengers.

**Table 1:** An overview of the MSSEG-2 dataset.

**Data**	**Modality**	**Median voxel size (mm)**	**Median shape**	**No. of total cases**	**No. with-new-lesion cases**	**No. without-new-lesion cases**
Training	FLAIR	(0.53, 0.98, 0.98)	(320, 256, 256)	40	29	11
Test	FLAIR	(0.65, 0.98, 0.98)	(280, 256, 256)	60	32	28
All	FLAIR	(0.60, 0.98, 0.98)	(297, 256, 256)	100	61	39

The third column indicates the median value of voxel size for each axis. The fourth column indicates the median number of voxels along each axis.

#### 4.1.2. Preprocessing

For each case, we first concatenate the two FLAIR images to form a 2-channel 3D image as the input. This is valid since the two FLAIR images are co-registered, and therefore, spatially aligned. The resulting image and its ground truth are then cropped with a minimal box filtering out zero regions. MSSEG-2 data are heterogeneous in the sense that the images may be acquired with different protocols in multiple institutes using different scanners, making intensity values greatly vary across patients and even across time points within the same patient. Therefore, we normalize each image channel-wise using a z-score to have intensities with zero mean and unit variance. Moreover, all the images and their ground truths are then resampled to the same voxel size of 1 mm^3^ using trilinear interpolation.

#### 4.1.3. Data augmentation

To reduce overfitting caused by data insufficiency and heterogeneity, it is crucial to perform an effective data augmentation workflow before feeding the data into the network.^P3^During training, the data preprocessing and augmentation are integrated into a single pipeline operating on a batch of 2 samples at each step on the fly. From each sample, we first crop a random 128 × 128 × 128 patch whose center lies within the foreground (i.e., new lesions) with a probability of 66%. Such an oversampling technique ensures that at least 66% of the patches contain some lesion, which in turn alleviates the class imbalance problem caused by the relatively small size of new lesions. The patches then undergo spatial transforms, including random affine and random flip along each spatial dimension, and intensity transforms, including random additive Gaussian noise, random Gaussian smoothing, random intensity scaling and shifting, random bias field, and random contrast adjusting. All the preprocessing operations and augmentation transforms are computed on CPU using the MONAI library ().

#### 4.1.4. Optimization

All networks are trained for 100,000 steps with a batch size of 2 (each patch is processed on one GPU) using AdamW optimizer with an initial learning rate of 1e − 5, weight decay of 1e − 2, and cosine annealing scheduler.^P3^Therefore, each network in training is fed by a total of 200,000 different patches of size 128 × 128 × 128. It is worth mentioning that since the training set consists of 40 subjects, there are 5,000 = 200,000/40 patches per subject, among which around 3,300 = 5,000 ×0.66 patches are expected to contain new lesions.

The loss $L_{\text{total}}$ is computed by incorporating the three deep supervision outputs and the corresponding downsampled ground truths, according to


(1) $$\begin{array}{l} L_{\text{total}}=\lambda_{0}L\left({\text{G}}_{0},{\text{P}}_{0}\right)+\lambda_{1}L\left({\text{G}}_{1},{\text{P}}_{1}\right)+\lambda_{2}L\left({\text{G}}_{2},{\text{P}}_{2}\right), \end{array}$$

where λ_0_ = 1, λ_1_ = 0.5, and λ_2_ = 0.25; **G**_*i*_ and **P**_*i*_ correspond to the deep supervision at resolution [128/(2^*i*^)]^3^; and the loss function L(·,·) is the sum of soft Dice (Milletari et al., 2016) and Focal loss (Lin et al., 2017), that is


(2) $$\begin{array}{l} L\left(\text{G},\text{P}\right)=L_{\text{Dice}}\left(\text{G},\text{P}\right)+L_{\text{Focal}}\left(\text{G},\text{P}\right), \end{array}$$

where


(3) $$\begin{array}{ll} L_{\text{Dice}}\left(\text{G},\text{P}\right) & =1-\frac{2\langle \text{G},\text{P}\rangle +\epsilon }{∥\text{G}{∥}^{2}+∥\text{P}{∥}^{2}+\epsilon },\\ L_{\text{Focal}}\left(\text{G},\text{P}\right) & =-\frac{1}{N}\langle \text{G},{\left(1-\text{P}\right)}^{\gamma }\log\left(\text{P}\right)\rangle , \end{array}$$

where **G** ∈ {0, 1}^*J*×*N*^ and **P**∈ [0, 1]^*J*×*N*^ represent the one-hot encoded ground truth and the predicted probability map for each voxel, respectively, with *J* denoting the number of segmentation classes and *N* denoting the number of voxels in the patch. The small constant ϵ = 10^−5^ is commonly used to smooth the soft Dice loss and avoid division by zero. The focusing parameter γ = 2 smoothly controls the rate at which well-classified voxels are suppressed in the Focal loss, and **1** denotes a *J* × *N* matrix of ones. The Focal loss has proved effective in tackling the class imbalance problem, which is present in the MSSEG-2 training set since the total volume of new lesions is generally much smaller than that of the background, and nearly one-third of the patients have no new lesions.

#### 4.1.5. Inference

A test image in the inference is first subjected to z-score intensity normalization and resampled to a voxel size of 1 mm^3^. The prediction is then made using a sliding window approach with a 50% overlap and a window size of 128 × 128 × 128 (which is equal to the patch size used in training).For a given voxel from overlapping windows, the mean of the predictions is simply taken as the final value (the SlidingWindowInferer module from MONAI was used to perform the sliding window inference). The resulting probability map is resampled back to the original voxel size and finally thresholded by 0.5 to obtain a binary segmentation map.

#### 4.1.6. Evaluation

The Dice score and Hausdorff Distance (HD) are used as metrics to assess the performance of segmentation forthe patients that have some new lesions in their ground truths. The Dice score measures the voxel-wise overlap between the ground truth and the prediction, defined as


(4) $$\begin{array}{l} \text{Dice}\left(\text{g},\text{y}\right)=\frac{2\overset{N}{\underset{n=1}{\sum }}g_{n}y_{n}}{\overset{N}{\underset{n=1}{\sum }}g_{n}+\overset{N}{\underset{n=1}{\sum }}y_{n}} \end{array}$$

where *g*_*n*_ ∈ {0, 1} and *y*_*n*_ ∈ {0, 1} represent the ground truth and the binary prediction for a voxel, respectively, and *N* is the number of voxels. Hausdorff Distance (HD) evaluates the distance between the boundaries of ground truth and prediction, computed according to:


(5) $$\begin{array}{l} \text{HD}(G,Y)=\max\{\underset{g\in G}{\max}\;\underset{y\in Y}{\min}\;\|g-y\|,\underset{y\in Y}{\max}\;\underset{g\in G}{\min}\;\|y-g\|\}, \end{array}$$

where *G* and *Y* denote the set of all voxels on the surface of ground truth and prediction, respectively.

Lesion-wise sensitivity (SEN), positive predictive value (PPV), and F_1_ score are used as metrics to quantify the detection rate of new lesions. Let **G** be the ground truth and **Y** be the prediction. To compute these lesion level metrics, we follow Commowick et al. (2018), according to which the connected components of **G** and **Y** (with a 18-connectivity kernel) are first extracted, and all new lesions smaller than 3 mm^3^ in size are removed, yielding new tensors $\tilde{\text{G}}$ and $\tilde{\text{Y}}$. The metrics are then defined as


(6) $$\begin{array}{l} \text{SEN}=\frac{\text{TP}}{\text{TP}+\text{FN}}, \end{array}$$

$$\begin{array}{l} \text{PPV}=\frac{\text{TP}}{\text{TP}+\text{FP}},\\ {\text{F}}_{1}=\frac{2\text{TP}}{2\text{TP}+\text{FP}+\text{FN}}, \end{array}$$

where TP, FP, and FN are the number of true positives, false positives, and false negatives, respectively, in the detection of new lesions (i.e., connected components). The rules by which a lesion is considered detected are explained in Commowick et al. (2018).

For cases without any new lesions in their ground truths, we use the following two metrics:

- The **N**umber of new **L**esions **P**redicted (NLP) by the algorithm. This is obtained by counting the number of connected components in the predicted segmentation.
- The **V**olume of new **L**esions **P**redicted (VLP) by the algorithm. This is obtained by simply multiplying the number of voxels in the predicted segmentation by the voxel volume.

All the metrics mentioned above were computed using animaSegPerfAnalyzer from the Anima toolbox (available at https://anima.irisa.fr/, RRID: SCR_017017 and RRID: SCR_01707).

## 5. Results and discussion

### 5.1. Quantitative evaluation

**Figure 4:**
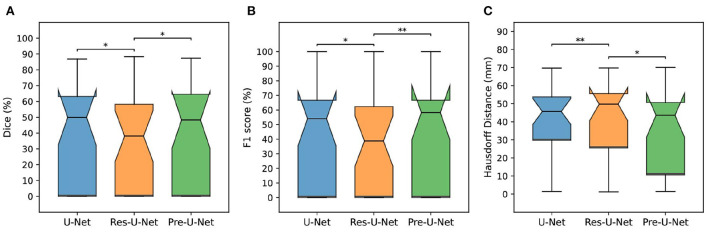
Comparison of different models on the MSSEG-2 test set. **(A–C)** Show box plots of Dice score (%), F1 score (%), and Hausdorff Distance (mm), respectively. The asterisks indicate how significantly a model score differs from those of the other baselines when using a pairwise Wilcoxon signed-rank test (*p-value < 0.05, **p-value < 0.01).

We performed five-fold cross-validation in all the experiments to estimate how capable our models are in generalizing to unseen data.The cross-validation results on the MSSEG-2 training set are reported in Table 2. For each network, we used an ensemble of the five models trained during the cross-validation on the training set for predicting the test set labels. The test results are reported in Table 3 and illustrated by notched box plots in Figure 4, where pairwise Wilcoxon signed-rank tests were used to identify the significant differences in the test scores of baselines.

**Table 2:** Results obtained by five-fold cross-validation on the MSSEG-2 training set.

**Model**	**No. of params**	**FLOPs**	**With-new-lesion cases**					**Without-new-lesion cases**	
			**Dice (%)↑**	**HD (mm)↓**	**SEN (%)↑**	**PPV (%)↑**	**F_1_ (%)↑**	**NLP↓**	**VLP (mm^3^)↓**
U-Net	28.7 M	1264.7 G	45.2(5.5)	**39.0**(14.1)	51.0 (12.1)	52.9 (6.1)	48.9(7.6)	0.1(0.2)	9.2(20.6)
Res-U-Net	28.9 M	1280.8 G	42.4(11.4)	46.4(15.9)	49.3 (22.9)	**60.6** (6.6)	49.9(14.8)	0.2(0.4)	4.0(9.0)
Pre-U-Net	28.9 M	1280.8 G	**45.6**(9.5)	40.1(13.2)	**54.5** (13.8)	53.8 (6.8)	**51.9**(11.3)	**0.0**(0.0)	**0.0**(0.0)

Symbols ↑ and ↓ indicate that a metric is desired to be higher and lower, respectively. The mean and standard deviation (SD) of a score across the folds are reported as “mean (SD).” The best results are in boldface.

**Table 3:** Results on the MSSEG-2 test set.

**Model**	**No. of params**	**FLOPs**	**With-new-lesion cases**					**Without-new-lesion cases**	
			**Dice (%)↑**	**HD (mm)↓**	**SEN (%)↑**	**PPV (%)↑**	**F_1_ (%)↑**	**NLP↓**	**VLP (mm^3^)↓**
U-Net	28.7 M	1264.7 G	38.9(31.1)	43.1(27.3)	45.2 (36.8)	51.2 (39.6)	45.3(35.7)	0.0(0.2)	0.4(2.3)
Res-U-Net	28.9 M	1280.8 G	34.9(29.5)	44.2(29.0)	43.6 (38.4)	38.4 (38.5)	33.7(33.1)	**0.0**(0.0)	**0.0**(0.0)
Pre-U-Net	28.9 M	1280.8 G	**40.3**(30.5)	**35.0**(22.3)	**47.5** (37.9)	**53.6** (38.3)	**48.1**(34.8)	0.0(0.2)	0.5(2.5)

Predictions were made using the five models from the cross-validation as an ensemble. Symbols ↑ and ↓ indicate that a metric is desired to be higher and lower, respectively. The mean and standard deviation (SD) of a score across patients are reported as “mean (SD).” The best results are in boldface.

Pre-U-Net was superior to all the other models in terms of both segmentation and detection performance for the test cases with some new lesions, achieving a Dice score of 40.3%, HD of 35.0, SEN of 47.5%, PPV of 53.6%, and F_1_ score of 48.1%. While having almost the same number of parameters and the same computational complexity (FLOPS), Pre-U-Net outperformed U-Net, the second-best baseline, and significantly outperformed Res-U-Net, with *p*-value < 0.05 for the Dice score, *p*-value < 0.01 for the F_1_ score, and *p*-value < 0.05 for HD.Overall, Pre-U-Net proved more effective than the other models at segmentation and detecting new lesions. Nevertheless, note that Pre-U-Net was only marginally superior to U-Net, and there was no statistically significant difference between the two models in terms of the segmentation or detection metrics.

Res-U-Net, with an NLP of 0.0 and VLP of 0.0, performed slightly better for the test cases that have no new lesions whereas Pre-U-Net is the winner in terms of validation scores. In fact, the differences in NLP and VLP scores are marginal, and all of our models are sufficiently accurate to detect no lesions (i.e., produce a segmentation map in which all elements are zero) for patients without any new lesions. Our team, LYLE, with the Pre-U-Net model (Ashtari et al., 2021a) was ranked first in the MSSEG-2 challenge in the two leaderboards based on the NLP and VLP metrics. All the four leaderboards (based on Dice, F_1_ score, NLP, and VLP metrics) and the patient-wise scores for each participating team can be found on https://portal.fli-iam.irisa.fr/msseg-2/challenge-day/.

### 5.2. Qualitative evaluation

Figure 1 presents qualitative comparisons of baselines. The top row exemplifies a patient with a single lesion that is detected by all the models. However, Pre-U-Net, with a patient-wise Dice score of 61.1%, yields a lesion that overlaps most with the lesion in the ground truth compared to U-Net with a patient-wise Dice score of 51.9% and Res-U-Net with a patient-wise Dice score of 36.9%.

Moreover, Pre-U-Net demonstrates superior performance in detecting new lesions. This capability is evidenced in the middle and bottom rows, where Pre-U-Net detects the two new lesions circled in yellow whereas U-Net and Res-U-Net fail to capture them. Note that as observed in the bottom row, Pre-U-Net, with a patient-wise Dice score of 68.3%, shows only a slight improvement in the segmentation performance over U-Net, with a patient-wise Dice score of 66.6%; however, Pre-U-Net indeed outperforms U-Net significantly when it comes to new lesion detection. Nevertheless, some new lesions are extremely challenging to detect even for experts, and all the models fail to capture them. For example, the lesion circled in blue on the ground truth (row 3 and column 3 in Figure 1) is detected by none of the models including Pre-U-Net.

Future work aims at improving new MS lesion detection, especially in the presence of such difficult lesions. This might include, for instance, incorporating the individual delineations of raters into our models. Indeed, in cases where there is more uncertainty due to a weaker consensus among raters (e.g., three raters delineated a set of voxels differently than the other one), our models are also more likely to result in false predictions. Moreover, we will investigate the possibility of transfer learning from a simpler lesion segmentation task with a bigger dataset for further tackling the data insufficiency and class imbalance problems faced in this work.

## 6. Conclusion

We devised a U-Net-like architecture consisting of pre-activation blocks, called Pre-U-Net, for longitudinal MS lesion segmentation. We successfully trained our models by using data augmentation and deep supervision, alleviating the problem of data insufficiency and class imbalance. The effectiveness of Pre-U-Net was evaluated in segmenting and detecting new white matter lesions in 3D FLAIR images on the MSSEG-2 dataset. Pre-U-Net achieved a Dice score of 40.3% and F_1_ score of 48.1%, outperforming the baselines, U-Net and Res-U-Net. In particular, Pre-U-Net is, as reflected by F_1_ scores, more effective than the baselines at detecting new lesions, and it is competitive with U-Net in terms of segmentation performance, as evidenced by Dice and HD scores.

## Data availability statement

Publicly available datasets were analyzed in this study. This data can be found at: https://portal.fli-iam.irisa.fr/msseg-2/data/.

## Ethics statement

The studies involving human participants were reviewed and approved by MICCAI 2021 MSSEG-2 challenge. The patients/participants provided their written informed consent to participate in this study.

## Author contributions

PA conceptualized, developed, validated the methodology of the study, wrote the first draft of the manuscript, and implemented the models. PA and BB contributed to the software development. SV acquired the funding. SV and DS-M supervised the work. All authors contributed to manuscript revision, read, and approved the submitted version.

## Funding

This research leading to these results has received funding from EU H2020 MSCA-ITN-2018: INtegrating Magnetic Resonance SPectroscopy and Multimodal Imaging for Research and Education in MEDicine (INSPiRE-MED), funded by the European Commission under Grant Agreement #813120. This research also received funding from the Flemish Government (AI Research Program). AI—KU Leuven Institute for AI, B-3000, Leuven, Belgium.

## Conflict of interest

The authors declare that the research was conducted in the absence of any commercial or financial relationships that could be construed as a potential conflict of interest.

## Publisher's note

All claims expressed in this article are solely those of the authors and do not necessarily represent those of their affiliated organizations, or those of the publisher, the editors and the reviewers. Any product that may be evaluated in this article, or claim that may be made by its manufacturer, is not guaranteed or endorsed by the publisher.
